# Providing emergency mental health support to Israeli civilians evacuated from their homes following the events of October 7th, 2023

**DOI:** 10.1186/s13584-025-00701-8

**Published:** 2025-06-26

**Authors:** Amit Yaniv-Rosenfeld, Ori Ganor, Ariel Gaon, Rinat R. Yedidya, Lior Azimi, Melanie Shmulevich, Shlomo Mendlvoic, Ido Lurie

**Affiliations:** 1 Shalvata Mental Health Center, Hod Hasharon, Israel; 2 Department of Psychiatry, Sackler School of Medicine, Tel Aviv University, Tel Aviv, Israel; 3 Hadekel Mental Health Clinic, Eilat, Israel

## Abstract

On October 7th, 2023, a deadly attack was launched on southern Israel from the Gaza Strip followed by major clashes along the Israel–Lebanon border. In the following days, approximately 2.5% of the Israeli population was evacuated from their homes, many of whom were directly affected by the violence Many evacuees were housed in Eilat, a small geographically peripheral city known for its holiday atmosphere in the southernmost part of Israel. The horrors of the terror attacks and the war, the unprecedented number of evacuees, and the highly limited mental health resources available in this remote city have combined to create an overwhelming demand for mental health services, which required the deployment of special measures. In this report from the field, we discuss our experiences in sending the first organized, organic teams to provide primary mental health support to the evacuees, with the supervision of Shalvata Mental Health Center, located over 300 km away from Eilat. Our experience highlights the need for proper preparation, planning, and practice for large-scale mental health support intervention in mass evacuation events and points to several successful and suboptimal practices for future deployment.

## Introduction

On October 7th, 2023, Hamas launched a deadly terror attack on southern Israel, followed by major clashes between Israel and Hezbollah along Israel’s northern border. Since then, the state of an ongoing war has led to devastating effects on civilians, including mass casualties, injuries, and displacement [5], [11], [16].

In the following days, about 250,000 Israeli civilians (2.5% of the Israeli population at that time), were evacuated from their homes to protect them from the dangers of the war, many of whom were directly affected by the violence (Homefront [14]. Most evacuees were temporarily accommodated in hotels and guest houses across the country, funded by the government. Nearly 50,000 of these evacuees arrived in the small peripheral city of Eilat, a holiday location in the southernmost part of Israel (~320 km from Tel Aviv), almost doubling the resident population.

To provide first mental aid and psycho-social support to the evacuees, the State of Israel was divided into 10 districts by the Ministry of Health (MoH) general director’s order (order #514942023, 16/10/2023). Each district was designated to serve a different number of evacuees, accommodated in nearby geographical areas. Given its remote peripheral location and limited mental health resources in the first place, the city of Eilat was designated as a separate district.

Eilat is the home of about 58,000 permanent residents (Homefront [14] and it provides very limited medical and mental health services, through a single hospital, Yoseftal [23], with 65 beds (less than 0.5% of the number of hospital beds in Israel, and the smallest in Israel) and a small community mental health clinic, HaDekel [12], with a total of 40 medical and para-medical personnel, some of whom work part-time. The nearest Israeli hospital outside Eilat is Soroka in Beer-Sheva, some 240 km away (Israeli [15]. The horrors of the mass terror attacks and the war, along with the unprecedented number of 50,000 evacuees, all combined into a sharp increase in the demand for mental health support in Eilat, a need which overwhelmed the city’s limited social and mental health system capacity, which had faced shortage of personnel and prolonged waiting lists even before the war.

To meet this extraordinary need for emergency mental health support, medical, paramedical, and psycho-social professionals, as well as other volunteers (e.g., complementary medicine therapists, administration), have all arrived at the city. The first hospital to send an organized organic team to provide mental health support was Shalvata Mental Health Center (SMHC). The deployment of the coordinated response started as soon as three days after the outbreak of war, on October 10th, 2023.

As it is crucial to study from past experiences of mass traumatic events [21], in this report from the field, we present and discuss our institute’s experience in quickly preparing and addressing the challenges and the potential lessons that could be learned for future events with mass traumatic exposure and extended population-level needs (e.g., due to conflict, natural disasters, public health emergencies). This brief report mainly focuses on organizing and delivering the primary mental health services, not characterizing the evacuees’ mental impact.

## About our institution

SMHC is located in central Israel and currently serves a total population of roughly 600,000 people [20]. It is affiliated with Tel-Aviv University and consists of four adult inpatient departments, a department of child and adolescent psychiatry, a day-care department, and a highly developed network of outpatient and community-based ambulatory services. In addition, it provides specialized services such as electroconvulsive therapy and a 24-hour emergency department, which serves over 3000 adult visits per year.

As of 2023, SMHC employed a total of 606 employees, of whom 85 psychiatrists (34 residents and 51 seniors), 159 nurses, 91 psychologists, 67 social workers, and 78 other psycho-social care providers (i.e., art therapists, occupational therapists, clinical criminologists).

## Operational model

In the weeks following the October 7th events, SMHC staff were involved in sending dedicated teams to Eilat. First, an official call for volunteers was sent to all relevant personnel via social media (i.e., WhatsApp group, Facebook). Overall, 251 people have volunteered with the following distribution: 40% psychologists, 25% psychiatrists, 25% social workers, and 10% others (including nurses, art therapists, occupational therapists, and administration). The volunteers first underwent a quick administrative process by the human resources (HR) department to ensure that they met the basic occupational requirements and appropriate personal liability insurance policies were secured to cover all of them. Then, designated training for supporting people with acute stress reaction (ASR), acute stress disorder (ASD), and anxiety was provided by senior personnel of the hospital, with extensive experience in post-traumatic reactions and delivering services in mental health crises (primarily by two of the authors, SM, IL). This training focused on psychological first aid (PFA, [3], ASR recognition, and crisis intervention techniques, as well as identifying and working with secondary traumatization. It included didactic lectures, interactive role-playing scenarios, and simulation exercises. Although a formal measurement of training was not conducted due to the time sensitive conditions, post-training informal feedback indicated a significant increase in confidence and readiness among the participants. Notably, participants mentioned group-level training as a favorable practice that allowed them to share and learn from prior experience. In addition, the supervision and guidance provided by senior hospital clinicians were mentioned as an essential formal dimension to the training that increased participants’ commitment.

The volunteers were divided into groups consisting of eight mental health professionals each, with a designated team leader. We deliberately avoided large groups in order to maintain high-quality service in SMHC, to minimize the risk of burnout and secondary traumatization, and to accommodate different logistical constraints and personal preferences. Each group was sent to Eilat for a period of 3-4 days. These groups have set up the needed infrastructure in the evacuees’ accommodations (a designated desk was set in 34 out of the 51 hotels and guest houses housing evacuees), coordinated with the relevant figures in the local mental health and social services systems, and have started to provide mental health support to those seeking it. Gradually, and with the understanding of the needs on the ground, a growing number of teams were sent simultaneously to Eilat, up to 40 volunteers at a time. The allocation of volunteers into teams and deployment was orchestrated by HR with the help of senior hospital clinicians, considering volunteers’ expertise, SMHC’s operational requirements, volunteers’ individual preferences, and the continually accumulated feedback from volunteers. This expert-based process was highly time-consuming, challenging, and potentially sub-optimal.

By the beginning of December 2023, and following the Israeli MoH plan and orders, the operation of the volunteer-based model was replaced by a stable, employee-based deployment of community mental health services by the four Health Funds in Israel.

## Outreach, interventions and challenges

We evaluated key outreach metrics, including the number of first mental aid sessions and support group participation, as well as qualitative feedback regarding the interventions and challenges provided or faced by volunteers and evacuees.

### Outreach

Overall, over 2600 sessions for 1591 evacuees took place during the first six weeks of deployment. In addition, more than 66 support group sessions consisting of 455 participants took place. Most sessions were provided for the adult evacuee population without a clear bias towards a specific age group or gender. Importantly, these figures should be considered as lower bounds for the actual outreach measures due to several challenges discussed below, primarily the request of many evacuees for their sessions not to be documented due to the stigma towards mental health services. Due to these challenges, additional measures were either not documented or inadequately recorded and, as a result, a comprehensive quantitative evaluation could not be performed.

### Interventions

Due to the conditions on the ground, volunteers’ capacity to systematically diagnose and document was limited. Nevertheless, volunteers reported that many evacuees were exhibiting emotional distress, grief, acute stress reactions, and acute stress disorders. PFA was provided to most evacuees to establish a sense of safety and comfort, stabilize emotional responses, gather information on needs and concerns, enhance access to social support, and provide practical assistance and coping information. Many evacuees reported a reduction in initial distress and better short-term adaptive functioning due to these sessions. While none of the evacuees required immediate inpatient care, a few were prescribed short-term medication such as selective serotonin reuptake inhibitors (SSRIs) or benzodiazepines. As such, the volunteers delivered immediate mental health support and performed follow-up care. In addition, hostility and a lack of trust towards official government representatives were noticeable. Most evacuees had no psychiatric history before the October 7th events. The opportunity to help the evacuees was reported as very fulfilling and important for the staff. However, it was also apparent that many volunteers experienced secondary traumatization and burnout, necessitating the implementation of on-site debriefing sessions and peer support networks. Of particular note, the deployment of staff members with diverse expertise, in organic teams, as well as their placement in the evacuees’ accommodations, were reported to be highly successful practices.

### Challenges

We encountered two key challenges: (1) Documentation. As noted above, many evacuees requested to avoid proper documentation of their interaction with us. This request, which was mostly met by the volunteers, posed a significant barrier towards proper record-keeping and subsequent quantitative data analysis. In addition, no standardized documentation protocol or computerized system were available for the volunteers before deployment, leading to sub-optimal, sometimes hand-written and paper-based record keeping. (2) Logistics. Notably, the division and scheduling of volunteers into teams and deployment required substantial time and effort, inherently leading to sub-optimal deployment at an increased effort. Furthermore, transportation and accommodation arrangements, as well as limited on-site resources for setting up the needed infrastructure was delayed, and may have decreased the effectiveness of the care provided.

## Discussion

### Mental health crises

Emergency and mass-traumatic exposure events, such as terrorism and war, can have a devastating impact on the mental well-being of the directly and indirectly affected civilian population [4, 6]. To meet the expected surge in the demand for mental health services, various models have been developed internationally, primarily focusing on internal organizational rearrangement, structural changes, and the utilization of community social networks [9], [18]. Unfortunately, these solutions do not seem appropriate for the case of mass evacuation into a small peripheral location with minimal available resources, such as the situation faced by Israel following the events of October 7th, 2023. To the best of our knowledge, existing models have yet to consider such an extreme setting explicitly.

Some parallels may be drawn with other large-scale mental health crisis responses, offering valuable insights into our setting. For instance, mental health initiatives among Syrian refugees have demonstrated the benefits of integrating mental health services into primary healthcare settings, thereby decentralizing care and reducing stigma [10, 17]. These community-based interventions, which include early PFA [3], have effectively mitigated acute stress reactions and fostered long-term resilience among displaced populations. Similarly, following the 2011 Great East Japan Earthquake, a stepped-care approach combining PFA, community outreach, and specialized care for severe cases proved successful in addressing a wide range of mental health needs [13]. Such international experiences highlight the importance of establishing sustainable, community-based mental health services that follow pre-event planning, robust training programs, and provide adaptable service models that can be quickly deployed during mental health emergencies, as suggested by the World Health Organization [22].

Israel has a long history of supporting civilians affected by terrorist attacks [1, 7, 8, 19]. These have led to the design and evolution of advanced operational models for quickly providing emergency mental health support [2]. The experiences accumulated following the October 7th events and the subsequent urgent need for mental health support by a large number of evacuees in a remote location present an opportunity for “building back better” [22].

### The volunteer-based model

Following the events of October 7th, a volunteer-based model was applied, with our institution playing a central role in the effort. Overall, 251 volunteers, encompassing the full range of personnel available in our institutions including senior physicians, residents, psychologists, social workers, nurses, and others, were sent to the evacuees’ accommodations, offering high-quality support over 6 weeks. Over 2600 individual sessions and 66 group sessions took place, primarily targeting grief and acute stress. The model was later replaced by a more stable community-based model enhanced by newly recruited employees. It is important to note alternative models—such as permanently staffed professional teams— could be considered in the future. However, given the large number of organically teamed mental health professionals willing to assist in such short order, especially in times of national turmoil and uncertainty, it seems that the deployment of the volunteer-based model following October 7th successfully overcame many of the typical pitfalls of such models: poor recruitment, lack of appropriate training and/or inadequate team coordination. Unfortunately, the lack of a structured framework to monitor and evaluate service utilization and outcomes further limits our ability to quantitatively evaluate the model. An important component of the model is the preceding designated training provided to the volunteers by the hospital’s senior staff. While most post-training informal feedback indicated favorable outcomes, we cannot conclusively determine the effectiveness of the training. The development and improvement of the training program in light our experience is planned for future work. An additional central component of the model is the allocation of volunteers to teams and deployment, a process that, in our case, could potentially been improved through more advanced technological solutions. While post-deployment feedback from the volunteers generally indicated satisfaction with their teams and deployment, the effectiveness of different team compositions or deployment schedules cannot be effectively evaluated. Nevertheless, it seems reasonable to expect more advanced allocation and scheduling technological solutions to better streamline this process.

### Recommendations

Our experience from the deployment of volunteer-based model following October 7th leads us to recommend the following measures to improve the response to future mass-evacuation events:

1. The MoH should initiate a systematic, structured retrospective evaluation (after-action review) of the emergency mental health support services provided in Eilat by SMHC and otherwise, as well as the community mental health professionals who replaced them since. This evaluation should integrate the lessons learned and best practices into a single comprehensive report.
2. The MoH should commission a designated training program informed by the retrospective evaluation suggested above. This training program should target diverse expertise and be offered to potential future volunteers.
3. Establish institute-level pre-trained organic teams comprising of diverse mental health professionals, retired personnel, and/or trained volunteers to enable rapid response. A team leader should be appointed to each team to allow swift communication.
4. The MoH should conduct periodical emergency drills and refresher training sessions to maintain readiness and improve future volunteers’ confidence and competence over time.
5. With the help of the technological private sector and/or research universities, standardized technological and logistical solutions should be developed, integrated and tested to streamline volunteer recruitment, coordination, deployment and documentation capabilities.
6. The protocols for the placement of evacuees in accommodations should be extended to include appropriate, pre-selected placements of emergency mental health stations within the evacuees’ accommodation or in their immediate surroundings.
7. Mental health institutes should enhance support mechanisms for volunteers, incorporating debriefing and structured mental health follow-ups during and following mass-trauma deployment.

## Data Availability

No datasets were generated or analysed during the current study.
